# How Does a Tumor Get Its Shape? MicroRNAs Act as Morphogens at the Cancer Invasion Front

**DOI:** 10.3390/ncrna6020023

**Published:** 2020-06-10

**Authors:** Catalin Vasilescu, Mihai Tanase, Dana Giza, Livia Procopiuc, Mihnea P. Dragomir, George A. Calin

**Affiliations:** 1 “Carol Davila” University of Medicine and Pharmacy, RO-020021 Bucharest, Romania; mihnea.p.dragomir@gmail.com; 2 Department of Surgery, Fundeni Clinical Hospital, RO-022328 Bucharest, Romania; 3 Polytechnic University of Bucharest, RO-060042 Bucharest, Romania; www.mihai@gmail.com; 4 Department of Family and Community Medicine, McGovern Medical School at The University of Texas Health Science Center at Houston, Houston, TX 77030, USA; danaelena.giza@gmail.com; 5 Guy’s and St. Thomas’ NHS Foundation Trust, London SE1 7EH, UK; livia.procopiuc@gmail.com; 6 Translational Molecular Pathology Department, The University of Texas MD Anderson Cancer Center, Houston, TX 77030, USA; 7 Center for RNA Interference and Non-coding RNAs, The University of Texas MD Anderson Cancer Center, Houston, TX, 77030, USA

**Keywords:** morphogenesis, invasion front, tumor border, microRNA, diffusion

## Abstract

The generation and organization of the invasion front shape of neoplasms is an intriguing problem. The intimate mechanism is not yet understood, but the prevailing theory is that it represents an example of morphogenesis. Morphogenesis requires the presence of specific molecules, known as morphogens (activators and inhibitors), which can diffuse and elicit dose-dependent responses in their target cells. Due to their ability to modulate most of the coding transcriptome, their well-established role in embryogenesis, and their capacity to rapidly move between neighboring and distant cells, we propose microRNAs as inhibitors that could shape the cancer invasion front. In order to explain the genesis of the tumor border, we use Alan Turing’s reaction diffusion model, refined by Meinhardt and Gierer. This assumes the existence of an activator called *a*, and an inhibitor called *h*, which we hypothesize could be a freely moving microRNA. We used the fractal dimension as a measure of tumor border irregularity. We observed that the change in fractal dimension associates with variations in the diffusion coefficient of the activator (*D_a_*) or the inhibitor (*D_h_*). We determined that the fractal dimension remains constant (i.e., the irregularity of the tumor border does not change) across a *D_h_* interval, which becomes narrower as *D_a_* rises. We therefore conclude that a change in fractal dimension occurs when the balance between *D_a_* and *D_h_* is disrupted. Biologically, this could be explained by a faulty distribution of the inhibitor caused by an abnormal density of the intercellular connection network. From a translational perspective, if experimentally confirmed, our observations can be used for a better diagnosis of cancer aggressiveness.

## 1. Introduction

The *cancer invasion front* of a primary neoplasm represents a spatially organized tumor site, where many aspects of cancer development and progression are regulated both at single- and collective-cell levels. Paracrine signaling between cancer cells and associated stromal cells is almost exclusively present at the invasive sites [1,2].

The cancer invasion front configuration correlates with the degree of tumor invasiveness, patient survival and prognosis [3]. New insights were gained into the clinical significance of tumor border shape through the application of fractal geometry techniques [4,5]. How the shape of cancer invasion front is generated is an intriguing problem. The intimate mechanisms are not yet understood, but the prevailing theory is that they represent an example of morphogenesis, a process that occurs in normal embryogenesis [6,7].

The concept of “morphogen” was first introduced by Alan Turing in 1952 in his search for an appropriate term to describe the emergence of structure and form during embryogenesis [8]. Turing postulated that a tissue may be a mass of cells within which molecules diffuse and participate in chemical reactions. These substances are called morphogens since they are the sole determinant of form in the given system. Turing’s morphogenic model discusses the way these biochemicals interact when they circulate across cells that communicate. Turing also proposed that the morphogen circulates by obeying the laws of diffusion.

Turing’s model was further refined by Meinhardt and Gierer. They describe biological pattern formation more completely by constructing a model that consists of two partial differential equations of reaction-diffusion type [9].

Further, Inui et al. propose a model wherein cells exposed to higher levels of morphogen activate a gene expression program distinct from that one induced in cells exposed to lower levels of the same signal [10]. Neighboring cells exposed to small differences in morphogen concentration can adopt markedly different fates. Cells ‘interpret’ positional values and adopt qualitatively different cell states depending on their positional values. This requires a *threshold-dependent mechanism* of interpretation, where cells with positional values above a certain threshold will enter one state, while cells with values below threshold will enter another. This concept (also known as the “*French flag model*”) described by Wolpert, opened the way to a new understanding of developmental patterning. Wolpert’s model defines positional information as being entirely independent of its subsequent interpretation. Development is a two-step process consisting of: (1) pattern formation based on positional information, and (2) the differentiation of cells according to the cell’s genome and developmental history [11].

It is still a matter of debate if cancer is best modelled at the single cell level or as a fundamentally tissue and organ phenomenon. Whether genetic mutations or epigenetic alterations play the biggest role is also a matter of debate [7]. The idea that cancer is a developmental disease is not new. In 2001, Potter proposed a hypothesis whereby tissue integrity and its disruption (disruption of morphostatic control) is permissive for loss of microarchitecture integrity and is a central event in carcinogenesis. This concept was termed by Potter morphostasis [6]. The same idea was used by van den Brink and Offerhaus to describe similar concepts along with the experimental evidence for morphostasis and specific agents that are specifically in relation to colon cancer [12]. The position within the host remains an important factor for tumorigenesis. The information that could be contained within the morphogenetic field is a prepattern—a scaffold that serves as a template, to some level of detail, for the tumor shape.

MicroRNAs (miRNAs) are 19–25 nucleotide long, endogenously produced, single stranded RNA molecules, which have mainly been proven to regulate gene expression at the post-transcriptional level. They achieve this through binding to specific sequences on the 3’ untranslated region (3’UTR) of their target mRNAs, called miRNA response elements (MREs) [13,14]. The interaction between the seed region of miRNAs and the MREs of mRNAs leads to the inhibition of translation and/or the degradation of the mRNA [13]. Furthermore, in recent years, several publications showed that miRNAs have additional unconventional functions, including the activation of gene expression [13], but these functions are exceptions. Hence, in our model, we will take into account only the main and best described function of miRNAs, post-transcriptional gene inhibition.

MiRNA-induced gene regulation can function in a combinatorial manner, if an mRNA transcript harbors numerous and distinct MREs, multiple miRNAs can inhibit its translation. Furthermore, each miRNA may repress up to hundreds of transcripts, and, thus, it is estimated that miRNAs regulate most of the coding transcriptome [15]. Since their discovery, experimental evidence has accumulated to prove that miRNAs are part of a complex machinery that regulates gene expression [16]. Epigenetic modifications, mutations, rearrangements, such as deletion, amplification, inversion, and chromosomal translocation, are observed to alter miRNA genes in cancer [17,18], and these alterations led to the discovery of miRNA functions in cancer development [17].

Cancer cells and their microenvironment, as pointed out by Hanahan and Weinberg, are characterized by ten essential features. These are: self-sufficiency in growth signals, insensitivity to anti-growth signals, apoptosis evasion, limitless replicative potential, sustained angiogenesis, tissue invasion and metastasis, deregulated cellular metabolism, evading immune destruction, genomic instability and tumor promoting inflammation [19]. Each of these processes are regulated by complex networks of miRNAs which means that dysregulation can render miRNAs as either tumor suppressors or oncogenes [20].

Morphogenesis requires the presence of specific molecules, known as morphogens, which can diffuse and elicit dose-dependent responses in their target cells. A morphogen is by definition any chemical agent able to cause or determine morphogenesis. Thus, both the activator and the inhibitor are morphogens. Their interaction drives the process of morphogenesis [9]. The presence of a morphogen concentration gradient across a cell range is essential for this model. The gradient establishment requires a localized morphogen secretion, diffusion, and clearance from the extracellular space. Herein, because the best described function of miRNAs is to down-regulate gene expression, we propose that miRNAs could be morphogenetic inhibitors. As previously mentioned, miRNAs act mainly as post-transcriptional regulators of their target mRNAs. A closer look at this phenomenon reveals that miRNA effects are modest: even when exogenously over-expressed, miRNAs can rarely down-regulate their targets by more than 50% [21], but these modest changes in gene expression can induce important biological effects [22]. This proves that miRNAs act as fine tuners of the coding transcriptome, making them an ideal candidate for the regulation of the morphogen pathway. Moreover, the first discovered miRNAs were shown to play important roles in the development of *C. elegans* larvae [23,24].

Levine et al. were the first advocate for the ability of miRNAs to play a role in morphogenesis by computing a mathematical model based on biological evidence. Their study shows that small RNAs may diffuse into regions with pre-existing mRNA concentrations and sharpen the interface between high-concentration and low-concentration regions [25].

One of the main characteristics of morphogens in the proposed model is their ability to move across the extra- and intracellular environment to reach their targets [26]. Experimental evidence supports that miRNAs exist both inside and outside the cell and their concentration varies between different body fluids and between different states of health and disease, proving that there is a regulated miRNA export and transport mechanism. Extracellular miRNAs are key elements of cell-to-cell communication and are not just the result of apoptosis or cell destruction.

The observation that extracellular miRNAs are resistant to the extremely abundant RNases found in human body fluids, led to the supposition that they travel in a protected fashion. Indeed, studies have revealed that miRNAs travel inside extracellular vesicles, especially in small membrane vesicles called exosomes (50–100 nm) [27]. Kosaka et al. found that miRNAs are first incorporated into exosomal particles, after which a surge of cellular ceramide stimulates the release of exosomes [28]. Further investigations revealed that extracellular miRNAs circulate in body fluids also bound to proteins, such as NPM1 and AGO2 [29], or lipoproteins, such as HDL [30].

Concerning the intracellular mobility, a high-resolution microscopy study by Pitchiaya et al. followed microinjected and fluorescent labelled miRNAs as they moved inside HeLa cells. They found the miRNAs to diffuse according to two Brownian patterns, which they hypothesize represent the two higher forms in which miRNAs organize themselves after they bind their target mRNA [31].

More recently, miRNAs were shown to move between cells through gap junctions, thus facilitating a rapid connexin-dependent cell-to-cell transfer that takes place in minutes and can influence the developmental fate of the receiving cells [32,33]. Adding to this, another mechanism on intercellular miRNA transport has been proposed, involving actin base cytoplasmic protrusions. These are called tunneling nanotubes (TNTs) and can connect various types of cells across distances of several hundred micrometers [34]. Given that their diameter is larger than that of the gap junctions, miRNAs and even mRNAs can easily circulate through TNTs [35,36]. Interestingly, Thayanithy et al. showed that TNTs mediate tumor stromal cross talk by allowing for transfer of oncogenic miRNAs [37].

The model we propose seeks to explain the genesis of the tumor border starting from Alan Turing’s reaction diffusion model, refined by Meinhardt and Gierer. This assumes the existence of an activator, called *a*, and an inhibitor, called *h*, which we hypothesize could be a freely moving miRNA. The conditions in our model is that the diffusion coefficient of *h*, called *D_h_* (i.e., the velocity with which the miRNA moves between cells) is greater than that of *a*, called *D_a_*. Given the in vivo and ex vivo studies cited above, it appears reasonable to assume that a small molecule such as miRNA can quickly transfer between cells, especially via TNTs acting as highways between cells [38,39].

**Figure 1:**
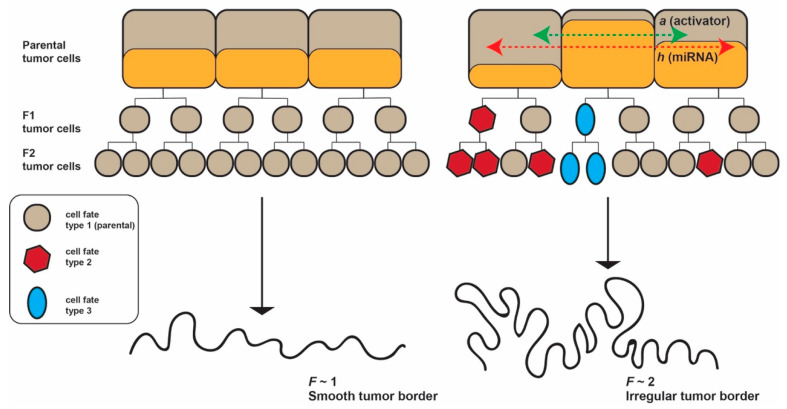
We hypothesize that miRNAs travel to neighboring cells and act as morphogenetic inhibitors in shaping the tumor border of epithelial cancers, and that the *diffusion coefficient* of miRNAs is probably a major factor influencing the invasion front configuration. Three different cell generations are represented: parental, F1 and F2. If the concertation of activator and inhibitor does not change in parental cells, the F1 and F2 cells preserve the same fate type and the tumor border is smooth. If the activator and inhibitor diffuse into neighboring parental cells, new cell fate types emerge and the tumor border becomes irregular. *F* = fractal dimension.

Taken together, our working hypotheses are: (i) miRNAs act as morphogenetic inhibitors in shaping the tumor border of epithelial cancers; and (ii) miRNAs exert their effect by diffusion in the tumor tissue and the diffusion coefficient of miRNAs is a major factor influencing the invasion front configuration (Figure 1).

## 2. Materials and Methods

### 2.1. Mathematical Model

These equations describe the concentrations of two substances, a self-enhancing activator and an inhibitor, the system therefore being called an activator–inhibitor system. The self-enhancement of the activator alone would lead to unlimited increase of the activator. Therefore, the activator, which is short-range autocatalytic, usually produces its own antagonist, a long-range inhibitor. 

**Figure 2:**
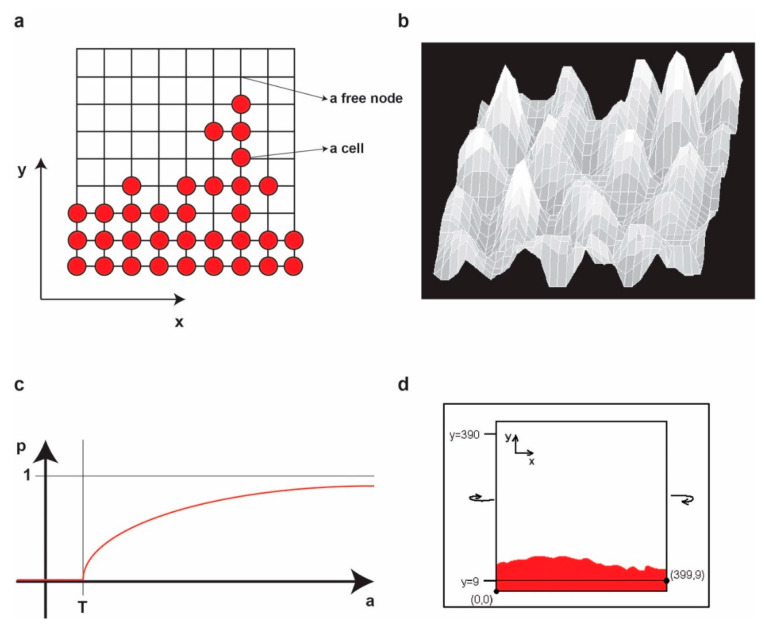
(**a**) An example of a small grid configuration. To each node (*x, y*) (either free or occupied) we associate the concentrations of *a* and *h* in (*x, y*). (**b**) An example of the ’landscape’ representation of the concentrations of *a* for some parameters at some moment in time. Higher values correspond to higher peaks. (**c**) The graph of *p(a)*. (**d**) The evolution of the populations of cells from the initial population inside the [0,0]–[399,9] box.

Let *L* be a regular lattice of elements called *nodes* (i.e., a regular squared grid of nodes). Each node on the lattice has integer coordinates (*x*, *y*). The nodes represent locations in the physical space in which a cell may be present or not. In the first case, we denote that the node is occupied by a cell, while, in the second case, we denote that the node is free. In addition, we attach to each node a pair of values (*a*, *h*) representing the concentration of activator (*a*) and inhibitor (*h*), respectively, in the node (Figure 2a).

The Meinhardt–Gierer differential equations of activator-inhibitor systems are [9]:

(1) $$\frac{\partial a}{\partial t}=D_{a}\Delta a+\rho_{a}\frac{a^{2}}{\left(1+k_{a}a^{2}\right)h}-\mu_{a}a+\sigma_{a}$$

(2) $$\frac{\partial h}{\partial t}=D_{h}\Delta h+\rho_{h}a^{2}-\mu_{h}h+\sigma_{h}$$

where Δ is the Laplace operator, *D_a_* and *D_h_* are the diffusion constants for *a* and *h,* respectively, *μ_a_* and *μ_h_* are the removal rates, *ρ_a_* and *ρ_h_* are the cross-reaction coefficients, *σ_a_* and *σ_h_* are basic production terms and *k_a_* is a saturation constant.

To apply the equations on our lattice, we write them in the following discrete form:

(3) $$a_{t+1}\left(x,y\right)=a_{t}\left(x,y\right)+D_{a}\left(\left(\sum_{\left(i,j\right)\in {\left\{-1,+1\right\}}^{2}}a_{t}\left(x+i,y+j\right)\right)-4\cdot a_{t}\left(x,y\right)\right)+\rho_{a}\frac{a_{t}{\left(x,y\right)}^{2}}{\left(1+k_{a}a_{t}{\left(x,y\right)}^{2}\right)h_{t}\left(x,y\right)}-\mu_{a}a_{t}\left(x,y\right)+\sigma_{a}$$

(4) $$h_{t+1}\left(x,y\right)=h_{t}\left(x,y\right)+D_{h}\left(\left(\sum_{\left(i,j\right)\in {\left\{-1,+1\right\}}^{2}}h_{t}\left(x+i,y+j\right)\right)-4\cdot h_{t}\left(x,y\right)\right)+\rho_{h}a_{t}{\left(x,y\right)}^{2}-\mu_{h}h_{t}\left(x,y\right)+\sigma_{h}$$

In this new setup, *t* is a natural number denoting the discrete time moment.

We fix a base concentrations pair (*A, H*) and we setup the initial concentrations of (*a, h*) on the lattice by:

(5) $$a_{0}\left(x,y\right)=A+\varepsilon_{a}\left(x,y\right)$$

(6) $$h_{0}\left(x,y\right)=H+\varepsilon_{h}\left(x,y\right)$$

where *ε_a_* (*x*, *y*) and *ε_h_* (*x*, *y*) each represent a small random variation (positive or negative) in an interval [-*ε*, +*ε*].

In our experiment, the lattice size is 400 by 400 nodes, *A* = *H* = 1.0, *ε* = 0.01.

We fix the parameters: *μ_a_* = 0.01, *μ_h_* = 0.02, *ρ_a_* = 0.01, *ρ_h_* = 0.02, *σ_a_* = 0.0, *σ_h_* = 0.0 and, *k_a_* = 0.0. For these particular values, the equations become:

(7) $$a_{t+1}\left(x,y\right)=a_{t}\left(x,y\right)+D_{a}((\sum_{\left(i,j\right)\in {\left\{-1,+1\right\}}^{2}}a_{t}\left(x+i,y+j\right))-4\cdot a_{t}\left(x,y\right))+\rho_{a}\frac{a_{t}{\left(x,y\right)}^{2}}{h_{t}\left(x,y\right)}-\mu_{a}a_{t}\left(x,y\right)$$

(8) $$h_{t+1}\left(x,y\right)=h_{t}\left(x,y\right)+D_{h}((\sum_{\left(i,j\right)\in {\left\{-1,+1\right\}}^{2}}h_{t}\left(x+i,y+j\right))-4\cdot h_{t}\left(x,y\right))+\rho_{h}a_{t}{\left(x,y\right)}^{2}-\mu_{h}h_{t}\left(x,y\right)$$

To avoid any artificial boundary conditions, we consider the lattice on a torus (i.e., we identify the left edge with the right edge of the lattice and we identify the top edge and bottom edge of the lattice) (Figure 2b,c).

In the following, we define the rules of cellular growth on the lattice. We fix two parameters: *T* (absolute grow threshold) and *R* (absolute rate of growth). In the experiment, we take *T* = 1.0 and *R* = 0.1.

We consider an initial population of living cells on the lattice. As stated earlier, the living cells correspond to the nodes (*x*, *y*) for which *c*(*x*, *y*) = 1.

At any iteration from time *t* to time *t* = 1 we compute the probability for a cell to divide by the following formula:

(9) $$p_{t}\left(x,\;y\right)=\{\begin{array}{c} 1-\frac{1}{1+R\cdot a_{t}\left(x,y\right)},\;\;if\;a_{t}\left(x,y\right)\geq T\\ 0,\;\;otherwise \end{array}$$

Practically, at time *t*, for any occupied node, we generate a random number between 0 and 1 and check if this number is less than *p_t_*(*x*, *y*). If this is the case, then the cell in that node will be considered to divide. However, it will not divide if there is no free node in its neighborhood (i.e., its left-right-upper-down adjacent nodes). On the other hand, if there are free nodes in its neighborhood, the division will occur and a new cell will be born in a randomly selected free node in this neighborhood (i.e., for one randomly selected free node in the neighborhood, say (*x* + *i*, *y* + *j*) for some (*i*, *j*) ∈ {−1, +1}^2^ we change the value of c(*x* + *i*, *y* + *j*) from 0 to 1).

In the experiment, we start from an initial population of living cells placed on a band on the bottom of the grid of height 10 (i.e., the box of coordinates [0,0]–[399,9] on the grid) representing the frontier of the tumor. However, the height is irrelevant, but it may be chosen only greater or equal to 2.

To restrict the growth below the *y* = 0 row (i.e., the *y* = 400 row as the lattice is on a torus), we will not permit the cells in the nodes (*x*, 0) to divide for any *x*, no matter what the value of *a_h_* (*x*, 0) is. It is obvious that this will not influence in any sense the aspect of the upper frontier (Figure 2d).

For a given pair of values for the parameters (*D_a_*, *D_h_*), we simulate the growth of the initial population of cells on the grid by applying the Meinhardt-Gierer equations in discrete form and the previously described rules of growth at each iteration. We stop the simulation either after 10,000 steps or if there is any cell in a node on the limiting line (i.e., with *y* = 390).

We run the experiment for every values of the pair (*D_a_*, *D_h_*) in the set: {0.002, 0.004, 0.006, …, 0.030} × {0.00, 0.02, 0.04, …, 0.30} and we evaluate the fractal dimension of the upper frontier of the final configuration of cells for each simulation. 

### 2.2. Fractal Dimension Analysis

We measure the smoothness/irregularity of a curve (i.e., tumor frontier) by its fractal dimension (F), a real number between 1 and 2. One might plausibly think (similarly to fuzzy logic) that the curve is smoother when the F is closer to 1 and the curve is more irregular (or less smoother) when F is closer to 2. This definition is consistent with how irregularity or complexity of a curve is understood in terms of F in literature [40]. We estimate F by using the standard compass method. More precisely, we first track the contour at the pixel-level and extract it as a 1-dimensional array of contiguous pixels and then we apply the compass method as described in [41].

### 2.3. Mean Square Displacement Analysis

The mean square displacement (MSD) is computed by averaging over the squares of the distances between the final and the initial positions of each particle.

MSD at a given time (*t*) is calculated as:

(10) $$MSD\left(t\right)=\frac{1}{N}\sum_{i=1}^{N}∥x^{\left(i\right)}\left(t\right)-x^{\left(i\right)}\left(0\right){∥}^{2}$$

where *N* is the number of particles and $x^{\left(i\right)}\left(0\right)$ is the initial position of *i*-th particle, and $x^{\left(i\right)}\left(t\right)$ is the position of the *i*-th particle at time t [42].

## 3. Results

### 3.1. The Tumour Border Is Shaped by the Diffusion Coefficient of the Morphogenetic Activator and Inhibitor

**Figure 3:**
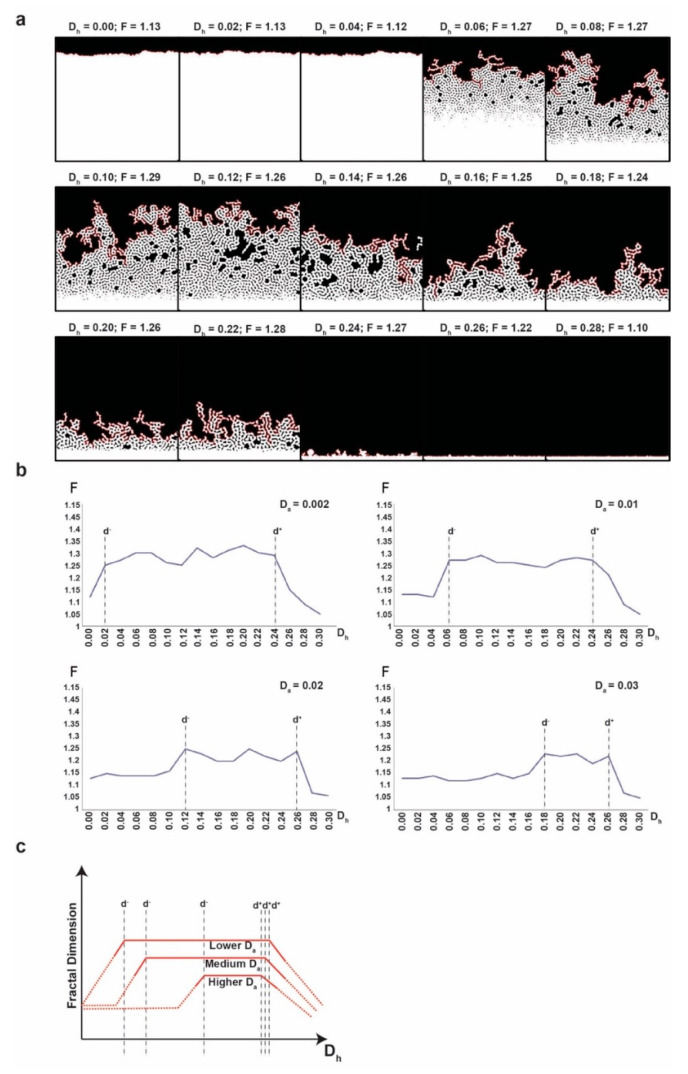
(**a**) Final configuration for *D_h_* = 0.00, 0.02, 0.04, …, 0.28 and *D_a_* = 0.01 in the left to right reading order. For each *D_h_* value, the corresponding fractal dimension (F) is presented. White colour depicts cells, black depicts empty space. (**b**) The fractal dimension (F) of the final configurations for *D_h_* = 0.00, 0.02, 0.04, …, 0.30 for four different values of *D_a_* (0.002, 0.01, 0.02 and 0.003). (**c**) The graphical representation of the dependence of the fractal dimension of *D_a_* and *D_h_*, illustrating the general phenomenon observed. With doted lines, we represent the lower values outside the intermediate interval for which we ignore the small variations, irrelevant for our conclusion.

We want to analyze the frontier of the cell population for various values of the diffusion coefficient of *D_a_* and *D_h_*. We observe that for some values the frontier is more complicated (i.e., has a higher F), while in some other cases it is almost smooth (i.e., F is close to 1). Naturally, we next want to see if there is a stable dependence between F and the values of *D_a_* and *D_h_*. Indeed, for any fixed chosen values of *D_a_* and *D_h_* we consistently obtain the same F of the frontier for a large number of stimulations. In Figure 3a we show one example of the final configuration obtained for each value of the diffusion coefficient *D_h_* in the set {0.00, 0.02, 0.04, …, 0.28}, while the value of *D_a_* = 0.01 remains fixed. In our simulation, we do not refer to the tumor volume as we do not simulate the entire tumor growth but only simulate the expansion front. We are only interested in the shape of the frontier. Thus, the difference in size of the cell mass for different values of *D_a_* and *D_h_* reflect the growth speed (or aggressiveness) of the tumor. In most types of cancer, the aggressiveness is higher when the frontier is irregular, while, in a few cancers, the situation is reversed [43,44]. The unbalanced morphogenesis from a biological perspective or degenerated morphogenesis from a mathematical perspective is caused either by a too low or a too high *D_h_* (i.e., outside the intermediate interval), respectively. The results suggest that the different aggressiveness in these two cases depends on the type of cancer, but this requires additional arguments and further investigation. 

Figure 3b shows different F values of the frontier obtained for the different values of *D_a_* and *D_h_*. We observe that the F of the frontier is higher when *D_h_* lies in the intermediate interval I = [d^-^, d^+^] and it is lower when *D_h_* lies outside this interval. Moreover, for values far from the intermediate interval, the final frontier loses its fractality completely (F = 1). On the other hand, higher values of *D_a_* tend to narrow the intermediate interval by shifting the left limit to the right (i.e., by increasing d^-^). At the same time, higher values of *D_a_* also tend to lower the maximum values of F. Additionally, we observe that outside the intermediate interval the frontier is significantly (and consistently) smoother as compared to how it is inside the intermediate interval (Figure 3c). We underline that Figure 3c is just an idealized representation of the dependence of F on *D_h_* for different values of *D_a_* to illustrate the general phenomenon that we observed. We are not interested in the small variations of values outside the intermediate interval or in particular at extreme values of *D_h_*. 

We detected that F of the tumor frontier is higher when *D_h_* lies in the intermediate interval, and higher values of *D_a_* narrow the interval. Hence, there is a balance between *D_h_* and *D_a_* that controls the tumor border. This balance depends most probably on the biological mechanisms that control the diffusion of the activator and inhibitor between tumor cells, such as TNTs, gap junctions, and exosomes. Disrupting this balance by blocking or increasing the transfer of miRNAs (inhibitors) between cells could change the shape of the tumor border. If our model is experimentally validated, exogenous miRNA transfer perturbations, that shift *D_h_* outside the interval, could be a future therapeutic intervention that inhibits tumor invasiveness. 

### 3.2. The Number of Intercellular Connections Affects the Fractal Dimension of the Tumor Frontier

**Figure 4:**
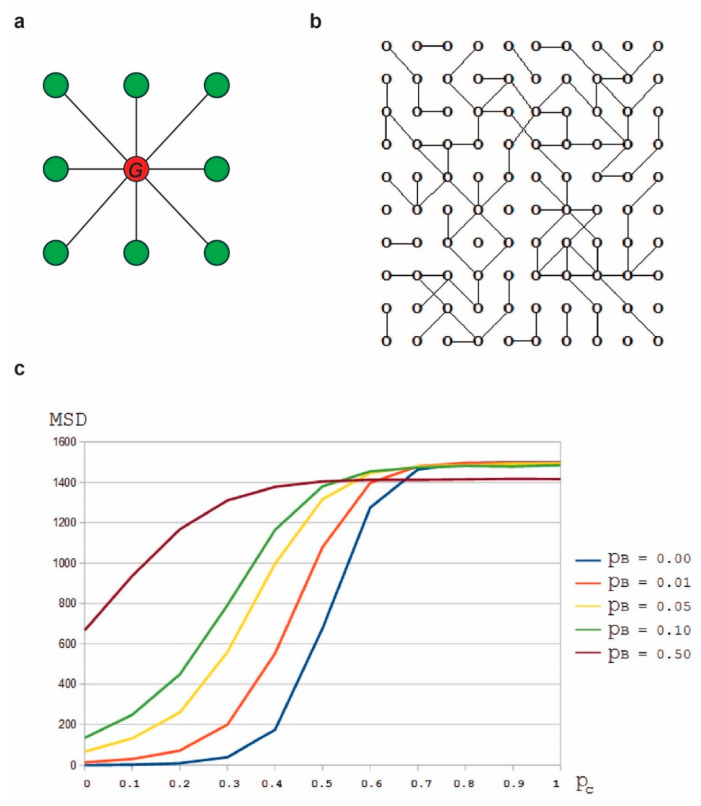
(**a**) Neighborhood of the red node consists of the set of green nodes. We connect the red node with each of the green nodes in its neighborhood with a probability $p_{c}$. We do this by generating a random number between 0 and 1 for each of the green nodes and check if this random number is less than $p_{c}$. If this is the case, then the red node and the green node are connected, otherwise they are not connected. (**b**) An example of a small cellular graph with $p_{c}=0.35$. (**c**) $\;MSD\left(p_{c},p_{B}\right)$ versus $p_{c}$ diagram, for different values of $p_{B}$ (the probability of a particle to reach a neighbor node by Brownian motion.

Going further, we introduce the element of intercellular mobility of small RNAs into our simulation. We model the cellular network as a graph and we study the diffusion on this graph to correlate the connectivity of the cellular network with the diffusion coefficient of the small RNA. In other words, we study the diffusion coefficient *D_h_* as a function of the probability $p_{c}$ that there is a link between two neighbor cells. We call these graphs “cellular graphs” and we define them in the following way: a cellular graph will be a graph G having as nodes the elements of the integral lattice L that we previously used for simulating the discrete Meinhardt–Gierer equations. We consider the neighborhood of each node as in Figure 4a. 

For each value of $p_{c}$ in the set {0, 0.1, 0.2, …0.9, 1} we generate 100 random instances of cellular graphs of size 2000 × 2000 nodes (with connectivity probability $p_{c}$) (Figure 4b). For the simulations, we also consider the probability $p_{B}$ of a particle to reach a neighbor node (not necessary connected to the initial node) by Brownian motion. This motion allows the particles not to get stuck in a subgraph that is not connected to the rest of graph. A higher value of $p_{B}$ will facilitate the diffusion of the particles.

The details on Brownian motion or any model of it are not relevant for our simulation as they are part of a lower level of abstraction than the level of abstraction that characterizes our simulation. We are using $p_{B}$ as a parameter that represents the probability for a particle to reach a non-adjacent neighbor node and point out that this can be achievable by Brownian motion, no matter how one would model it.

For each pair $\left(p_{c},p_{B}\right)$, we follow the random walk of 10,000 initially randomly placed particles (in nodes within a central area) for 1000 iterations. We compute the mean square displacement $MSD\left(p_{c},p_{B}\right)$ corresponding to each value of $p_{c}$. The mean square displacement $MSD\left(p_{c},p_{B}\right)$ is equivalent to the diffusion coefficient $D_{h}\;$(Figure 4c).

We observe that the mean square displacement grows with $p_{c}$. It means that the small RNA diffusion coefficient ($D_{h}$) grows with connectivity of the cellular network. Therefore, by fixing the value of $D_{a}$ and rephrasing the conclusions of our experiment with the discrete Meinhardt–Gierer equations, we conclude that the fractal dimension of the frontier is higher when the connectivity of the cellular network lies in a specific intermediate interval and it is lower when the connectivity lies outside this interval. In other words, too few or too many intercellular connections lead to a loss of fractality (i.e., complexity) of the frontier.

## 4. Discussion

The tumor edge and its adjacent peritumoral tissue are characterized by a dynamic process with a variable extent of cell rows identified at the invasion front. We aimed to investigate the mechanisms generating the patterns of the epithelial–stromal interface in an attempt to evaluate the invasiveness of the tumor, which could have further implications in the prognosis and orientation of the therapeutic strategies.

Tumor invasion involves an excess of proliferative, anti-apoptotic, and pro-migratory signals by cancer cells. Collective cell motion emerges from the forces and mechanisms that regulate individual cell motion or cellular communications via signaling between neighboring cells or long-range signals, such as diffusible morphogen gradients. 

Using Turing’s models of morphogen signaling transduction, we explored the mechanisms of tumor pattern formation. The context is like that of pattern formation on a growing biological surface modelled by reaction-diffusion equations following the classical paper of Turing [8]. Such equations exhibit a diffusion-driven instability of spatially uniformed structures, leading to spatially non-uniformed patterns such as that encountered in tumorigenesis.

Morphogenesis first ensued as the answer to a puzzling question: how do so many cell types arise from a single totipotent cell using only a limited array of signaling molecules? In the morphogen model, that answer lies in concentration gradients. The morphogenetic activator emanates from its source and diffuses across a certain cell range. This leads to cells lying farther from the morphogenetic activator source being subjected to a lower activator concentration than the ones nearer to the activator source. However, this variation is not sufficient, since experimental evidence has proven that even slight variations in morphogenetic activator concentration trigger dramatically different cell responses that ultimately lead to clear boundaries across tissues [7,11]. 

In keeping with the reaction-diffusion model imagined by Turing, i.e., a model involving an activator able to diffuse according to gas diffusion laws in physics and then to be consumed in a specific reaction inside a target cell, Meinhardt and Gierer refined a system where Turing’s activator was modulated by an inhibitor. This inhibitor could diffuse faster than the activator and it would contribute to steep drops in activator concentrations.

Which is the factor that translates the small concentration gradient into a net choice of cellular fates? The answer could be miRNAs. Levine et al. were the first to develop a mathematical model explaining how miRNAs could be inhibitors of early embryogenesis [25].

Mathematical modelling may bring important insight on tumor progression, help to explain experimental and clinical observations and to help assess optimal treatment strategies. We focused on a specific class of inductive pattern formation mechanisms, which depend on morphogen gradients, defined as the concentration fields of chemicals that act as dose-dependent regulators of cell signaling and gene expression. Our working hypothesis was that the morphological evolution of a growing solid tumor, the process of generating tumor patterns, is similar to that encountered in morphogenesis. Furthermore, we proposed that miRNAs act as morphogenesis inhibitors in shaping the tumor border of epithelial cancers.

The diffusion coefficient of miRNAs is probably a major factor influencing the invasion front configuration. We believe that miRNAs, this class of morphogen-like molecules, have a primary role in functional crosstalk among heterogeneous cells and in the maintenance of adult epithelia. 

The term morphostasis was described as the maintenance of tissue microarchitecture in a manner analogous to the role of a morphogen in morphogenesis [6]. The disruption of morphostatic control induces the loss of microarchitecture integrity and is a principal event in carcinogenesis. Therefore, disorders in miRNAs concentration might be manifestations of the loss of morphostasis and therefore a hallmark of tumorigenesis.

As previously mentioned, diffusion helps establish a concentration gradient. Experimental evidence exists for the extracellular transport of miRNA as well as the intracellular diffusion of miRNA. For the latter, Pitchiaya et al. used a high-resolution method to trace the fate and movement of miRNAs once injected into a cell. Performing diffusion measurements of fluorophore-labelled miRNAs (let-7a-1 and cxcr4) in HeLa cells, they identified two distinct Gaussian distributions of microscopic diffusion constants, implying two patterns of migration corresponding to two miRNA-containing complexes. Their imaging system followed one, or even more miRNAs forming large complexes with either messenger ribonucleoproteins or processing bodies and then being released from these complexes [31].

Connor et al. bring experimental evidence to our hypothesis by showing that cancer cells form TNTs conduct miRNAs in a horizontal fashion from the cancer cell to the surrounding stromal cells. They also demonstrate that the receiving stromal cells will exhibit a pathological phenotype, thus forming the molecular basis of understanding the metastatic potential of cancer cells [45].

## 5. Conclusions

Our model confirms part of the hypothesis that the morphogenetic activator and inhibitor play a role in tumor growth and pattern formation. We found that the concentration gradient of the inhibitor is essential. We used a computer simulation of the fractal dimension (F) of the tumor border as a measure of tumor border irregularity. We observed the change in F associated with variations in activator gradient (*D_a_*) or inhibitor gradient (*D_h_*). We concluded that F remains constant—i.e., the irregularity of the tumor border does not change across a *D_h_* interval that becomes narrower as *D_a_* rises. We therefore consider that a change in F occurs when the balance between *D_a_* and *D_h_* is disrupted. Biologically, this could be explained by a faulty distribution of the inhibitor. 

Experimental studies are necessary to further confirm our hypothesis. The identification of a pool of target miRNAs through high-throughput sequencing techniques and their search through hybridization studies in the tumor border would allow for the establishment of a correlation between the tumor border’s F and the number of identified miRNAs.

## References

[1] Karagiannis G.S., Petraki C., Prassas I., Saraon P., Musrap N., Dimitromanolakis A., Diamandis E.P. (2012). Proteomic signatures of the desmoplastic invasion front reveal collagen type XII as a marker of myofibroblastic differentiation during colorectal cancer metastasis. Oncotarget.

[2] Karagiannis G.S., Poutahidis T., Erdman S.E., Kirsch R., Riddell R.H., Diamandis E.P. (2012). Cancer-associated fibroblasts drive the progression of metastasis through both paracrine and mechanical pressure on cancer tissue. Mol. Cancer Res..

[3] Hase K., Shatney C., Johnson D., Trollope M., Vierra M. (1993). Prognostic value of tumor “budding” in patients with colorectal cancer. Dis. Colon. Rectum..

[4] Calin G.A., Vasilescu C., Negrini M., Barbanti-Brodano G. (2003). Genetic chaos and antichaos in human cancers. Med. Hypotheses.

[5] Vasilescu C., Olteanu M., Flondor P. (2012). Fractal-like kinetics, a possible link between preconditioning and sepsis immunodepression. On the chemical basis of innate immunity. Chirurgia.

[6] Potter J.D. (2001). Morphostats: A missing concept in cancer biology. Cancer Epidemiol. Biomark. Prev..

[7] Levin M. (2012). Morphogenetic fields in embryogenesis, regeneration, and cancer: Non-local control of complex patterning. Biosystems.

[8] Turing A. (1952). The chemical basis of morphogenesis. Philos. Trans. R. Soc. Lond..

[9] Gierer A., Meinhardt H. (1972). A theory of biological pattern formation. Kybernetik.

[10] Inui M., Montagner M., Piccolo S. (2012). miRNAs and morphogen gradients. Curr. Opin. Cell Biol..

[11] Wolpert L. (1969). Positional information and the spatial pattern of cellular differentiation. J. Biol..

[12] Van den Brink G.R., Offerhaus G.J. (2007). The morphogenetic code and colon cancer development. Cancer Cell.

[13] Dragomir M.P., Knutsen E., Calin G.A. (2018). SnapShot: Unconventional miRNA Functions. Cell.

[14] Fabbri M., Girnita L., Varani G., Calin G.A. (2019). Decrypting noncoding RNA interactions, structures, and functional networks. Genome Res..

[15] Friedman R.C., Farh K.K., Burge C.B., Bartel D.P. (2009). Most mammalian mRNAs are conserved targets of microRNAs. Genome Res..

[16] Dragomir M., Mafra A.C.P., Dias S.M.G., Vasilescu C., Calin G.A. (2018). Using microRNA networks to understand cancer. Int. J. Mol. Sci..

[17] Calin G.A., Dumitru C.D., Shimizu M., Bichi R., Zupo S., Noch E., Aldler H., Rattan S., Keating M., Rai K. (2002). Frequent deletions and down-regulation of micro- RNA genes miR15 and miR16 at 13q14 in chronic lymphocytic leukemia. Proc. Natl. Acad. Sci. USA.

[18] Lages E., Ipas H., Guttin A., Nesr H., Berger F., Issartel J.P. (2012). MicroRNAs: Molecular features and role in cancer. Front. Biosci..

[19] Hanahan D., Weinberg R.A. (2011). Hallmarks of cancer: The next generation. Cell.

[20] Dragomir M.P., Kopetz S., Ajani J.A., Calin G.A. (2020). Non-coding RNAs in GI cancers: From cancer hallmarks to clinical utility. Gut.

[21] Hausser J., Zavolan M. (2014). Identification and consequences of miRNA-target interactions—Beyond repression of gene expression. Nat. Rev. Genet..

[22] Bartel D.P. (2018). Metazoan MicroRNAs. Cell.

[23] Lee R.C., Feinbaum R.L., Ambros V. (1993). The C. elegans heterochronic gene lin-4 encodes small RNAs with antisense complementarity to lin-14. Cell.

[24] Wightman B., Ha I., Ruvkun G. (1993). Posttranscriptional regulation of the heterochronic gene lin-14 by lin-4 mediates temporal pattern formation in C. elegans. Cell.

[25] Levine E., McHale P., Levine H. (2007). Small regulatory RNAs may sharpen spatial expression patterns. PLoS Comput. Biol..

[26] Vasilescu C., Tanase M., Dragomir M., Calin G.A. (2016). From mobility to crosstalk. A model of intracellular miRNAs motion may explain the RNAs interaction mechanism on the basis of target subcellular localization. Math. Biosci..

[27] Chevillet J.R., Kang Q., Ruf I.K., Briggs H.A., Vojtech L.N., Hughes S.M., Cheng H.H., Arroyo J.D., Meredith E.K., Gallichotte E.N. (2014). Quantitative and stoichiometric analysis of the microRNA content of exosomes. Proc. Natl. Acad. Sci. USA.

[28] Kosaka N., Iguchi H., Hagiwara K., Yoshioka Y., Takeshita F., Ochiya T. (2013). Neutral sphingomyelinase 2 (nSMase2)-dependent exosomal transfer of angiogenic microRNAs regulate cancer cell metastasis. J. Biol. Chem..

[29] Turchinovich A., Burwinkel B. (2012). Distinct AGO1 and AGO2 associated miRNA profiles in human cells and blood plasma. RNA Biol..

[30] Vickers K.C., Palmisano B.T., Shoucri B.M., Shamburek R.D., Remaley A.T. (2011). MicroRNAs are transported in plasma and delivered to recipient cells by high-density lipoproteins. Nat. Cell Biol..

[31] Pitchiaya S., Androsavich J.R., Walter N.G. (2012). Intracellular single molecule microscopy reveals two kinetically distinct pathways for microRNA assembly. EMBO Rep..

[32] Lemcke H., Voronina N., Steinhoff G., David R. (2017). Analysis of the Gap Junction-dependent Transfer of miRNA with 3D-FRAP Microscopy. J. Vis. Exp..

[33] Valiunas V., Polosina Y.Y., Miller H., Potapova I.A., Valiuniene L., Doronin S., Mathias R.T., Robinson R.B., Rosen M.R., Cohen I.S. (2005). Connexin-specific cell-to-cell transfer of short interfering RNA by gap junctions. J. Physiol..

[34] Dupont M., Souriant S., Lugo-Villarino G., Maridonneau-Parini I., Verollet C. (2018). Tunneling nanotubes: Intimate communication between myeloid cells. Front. Immunol..

[35] Climent M., Quintavalle M., Miragoli M., Chen J., Condorelli G., Elia L. (2015). TGFbeta triggers miR-143/145 transfer from smooth muscle cells to endothelial cells, thereby modulating vessel stabilization. Circ. Res..

[36] Haimovich G., Ecker C.M., Dunagin M.C., Eggan E., Raj A., Gerst J.E., Singer R.H. (2017). Intercellular mRNA trafficking via membrane nanotube-like extensions in mammalian cells. Proc. Natl. Acad. Sci. USA.

[37] Thayanithy V., Dickson E.L., Steer C., Subramanian S., Lou E. (2014). Tumor-stromal cross talk: Direct cell-to-cell transfer of oncogenic microRNAs via tunneling nanotubes. Transl. Res..

[38] Rustom A., Saffrich R., Markovic I., Walther P., Gerdes H.H. (2004). Nanotubular highways for intercellular organelle transport. Science.

[39] Lou E., Gholami S., Romin Y., Thayanithy V., Fujisawa S., Desir S., Steer C.J., Subramanian S., Fong Y., Manova-Todorova K. (2017). Imaging tunneling membrane tubes elucidates cell communication in tumors. Trends Cancer.

[40] Peitgen H.O., Jürgens H., Saupe D. (2004). Chaos and Fractals.

[41] Mandelbrot B.B. (1982). The Fractal Geometry of Nature.

[42] Frenkel D., Smit B. (2001). Understanding Molecular Simulation.

[43] Lennon F.E., Cianci G.C., Cipriani N.A., Hensing T.A., Zhang H.J., Chen C.T., Murgu S.D., Vokes E.E., Vannier M.W., Salgia R. (2015). Lung cancer-a fractal viewpoint. Nat. Rev. Clin. Oncol..

[44] Landini G., Rippin J.W. (1996). How important is tumour shape? Quantification of the epithelial-connective tissue interface in oral lesions using local connected fractal dimension analysis. J. Pathol..

[45] Connor Y., Tekleab S., Nandakumar S., Walls C., Tekleab Y., Husain A., Gadish O., Sabbisetti V., Kaushik S., Sehrawat S. (2015). Physical nanoscale conduit-mediated communication between tumour cells and the endothelium modulates endothelial phenotype. Nat. Commun..

